# FOXP3: A Player of Immunogenetic Architecture in Lung Cancer

**DOI:** 10.3390/genes15040493

**Published:** 2024-04-15

**Authors:** Iwona Ziółkowska-Suchanek, Magdalena Żurawek

**Affiliations:** Institute of Human Genetics, Polish Academy of Sciences, Strzeszyńska 32, 60-479 Poznań, Poland; magdalena.zurawek@igcz.poznan.pl

**Keywords:** FOXP3, regulatory T cells, FOXP3 expression, tumor-infiltrating FOXP3-lymphocytes, tumor microenvironment, non-small cell lung cancer

## Abstract

The transcription factor forkhead box protein 3 (FOXP3) is considered to be a prominent component of the immune system expressed in regulatory T cells (Tregs). Tregs are immunosuppressive cells that regulate immune homeostasis and self-tolerance. FOXP3 was originally thought to be a Tregs-specific molecule, but recent studies have pinpointed that FOXP3 is expressed in a diversity of benign tumors and carcinomas. The vast majority of the data have shown that FOXP3 is correlated with an unfavorable prognosis, although there are some reports indicating the opposite function of this molecule. Here, we review recent progress in understanding the FOXP3 role in the immunogenetic architecture of lung cancer, which is the leading cause of cancer-related death. We discuss the prognostic significance of tumor FOXP3 expression, tumor-infiltrating FOXP3-lymphocytes, tumor FOXP3 in tumor microenvironments and the potential of FOXP3-targeted therapy.

## 1. Introduction

Regulatory T cells were initially discovered over two decades ago by Sakaguchi et al. in a distinct model of murine autoimmunity [1]. In 2001, human Tregs were identified in the thymus and peripheral blood of healthy individuals [2]. However, they are preferentially called regulatory cells instead of suppressor T cells; their primary and the most important function is to suppress or downregulate the induction of effector T cells. Treg cells are able to control the cell fate of self-reactive T cells and determine long-term tolerance. In humans and mice, Treg cells are characterized by the high expression of the IL-2 receptor alpha chain (IL-2Rα and CD25) and the expression of the transcription factor FOXP3, which is crucial for their development, function and stability [3]. Inactivating mutations in FOXP3 lead to severe autoimmunity with a scurfy phenotype in mice and Immunodysregulation Polyendocrinopathy Enteropathy X-linked (IPEX) syndrome in humans, which is manifested by immune dysregulation, polyendocrinopathy and enteropathy [4,5]. Moreover, the ablation of conditional FOXP3 allele Treg cells results in the generation of effector T cells, which cause inflammatory tissue lesions [6]. On the contrary, enhancing the expression of endogenous FOXP3 results in the potent regulatory function of CD4+ T cells [7]. Therefore, stable FOXP3 expression in mature Treg cells is essential for maintaining the transcriptional and functional program established during Treg development.

FOXP3 has been found to express abnormally in lung squamous cell carcinoma and lung adenocarcinoma, suggesting that this gene plays an oncogene role [8]. FOXP3+ Tregs are present in the tumor microenvironment (TME) where they promote tumor development and progression by the cytokine releasing and suppressing activity of cytotoxic T cells [9]. It was indicated that the high infiltration of FOXP3+ TILs in the TME is associated with an unfavorable prognosis in lung cancer [10,11,12,13].

FOXP3 is an essential element of complex mechanisms that modulate the immune response and consequently impose tumor behavior. In this review, we summarize the recent data regarding FOXP3 expression in lung cancer cells, spatial architecture of tumor-infiltrating FOXP3+ T cells, prognostic significance of FOXP3+ TILs, FOXP3 impact on lung cancer stemness and FOXP3+ TILs in the context of cancer therapy.

## 2. Lung Cancer Epidemiology

**Figure 1:**
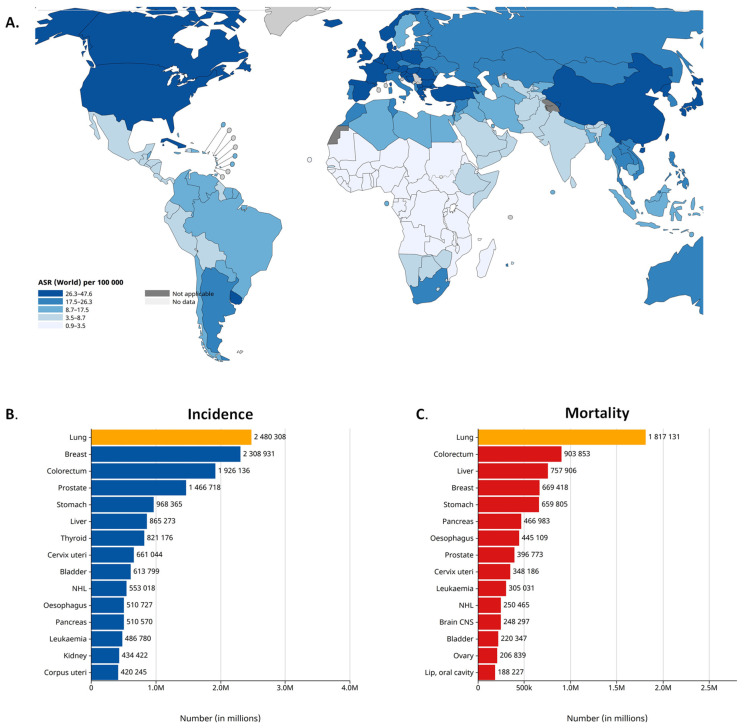
Lung cancer epidemiology. (**A**). Lung cancer incidence in 2022, both sexes. Age-Standardized Rate (ASR) per 100,000. (**B**). Lung cancer incidence in 2022 (world), both sexes. (**C**). Mortality caused by lung cancer in 2022 (world), both sexes. Source: International Agency for Research on Cancer, GLOBOCAN 2022 database [14,21].

Worldwide, lung cancer is the second most common cancer and the leading cause of cancer-related death. According to the latest GLOBOCAN 2020 estimates, lung cancer was diagnosed among 2.2 million new cases and caused 1.8 million deaths in 2020. Lung cancer is the leading cause of cancer morbidity and mortality in men, whereas, in women, it ranks third for incidence [14,15] (Figure 1). According to its histologic characteristics, lung cancer is divided into two categories, i.e., small-cell lung cancer (SCLC) and non-small-cell lung cancer (NSCLC), the latter of which is further divided [16]. NSCLC accounts for more than 80% of all lung cancer cases. As reported by the Surveillance, Epidemiology and End Results registry in the US, the incidence of NSCLC is 42.6/100,000 of the population [17]. The three main subtypes of NSCLC include adenocarcinoma (40% of lung cancers), squamous cell carcinoma (25%) and large-cell carcinoma (10%). Adenocarcinoma is the most common histologic subtype of lung cancer in men and women [18]. Although nowadays there are many new approaches for lung cancer therapies, the survival of patients with lung cancer at 5 years after diagnosis is only 10% to 20% in most countries among those diagnosed from 2010 to 2014 [14]. The 5-year relative survival rate varies markedly for patients diagnosed at the local stage (61%), regional stage (34%) or distant stage (7%) [19]. The prognosis of NSCLC patients remains poor because the majority of cases are diagnosed at the metastatic stage. The most frequent sites of lung cancer metastasis are the brain (20–40% of cases), the bone (39%) and the liver (16.3%). NSCLC contributes to approximately 50% of all brain metastases that occur in cancer patients [20].

## 3. Mutations/Genetic Variants in the FOXP3 Gene in NSCLC

Among the most frequently detected oncogenic mutations in NSCLC patients are *EGFR*-activating mutations, occurring in 10–20% of Caucasians and above 50% in the Asian population, based on the COSMIC database [22] and *KRAS* (Kirsten rat sarcoma virus) mutations with a frequency of around 30% [23]. The current landscape of driver mutations in NSCLC comprises alterations in *MET* (anaplastic lymphoma kinase), *c-ROS1* (receptor tyrosine kinase), *BRAF* (*v-raf* murine sarcoma viral oncogene homolog B), *NTRK1* (neurotrophic receptor tyrosine kinase), *HER2* (human epidermal growth factor 2) and *NGR1* (neuregulin-1) [24].

Based on the available literature data, there was not a well-established association between *FOXP3* mutations and NSCLC. It is known that mutations in the *FOXP3* gene can lead primarily to autoimmune disorders, with the most well-known being IPEX syndrome [25,26]. For patients with NSCLC, it is thought that FOXP3 as a master regulator of T cell development and function may modulate susceptibility to NSCLC. Recently, the analysis of FOXP3, with the usage of the cBioPortal database, revealed that gene alterations in FOXP3 occurred in 2.2% of the NSCLC samples [27].

Several *FOXP3* single-nucleotide polymorphisms (SNPs) were detected and its role in cancer susceptibility was investigated. The T allele in rs3761549 (T/C) *FOXP3* was correlated with a susceptibility to lung carcinoma among the Iran population [28], whereas the A allele of rs3761548 significantly increased the NSCLC risk (OR = 2.32 and 95% CI = 1.736–3.102) in Chinese patients [29]. The association of three *FOXP3* functional SNPs (rs3761548, rs3761549 and rs2280883) and cancer risk was performed by an updated meta-analysis. It revealed that rs3761548 contributes to an increased risk of cancer in the overall population, while no significant differences were observed in individual cancer groups, including breast, lung, hepatocellular, colorectal and thyroid cancer [30]. Another meta-analysis including the same three SNPs in six cancer groups showed that in the case of rs3761548 (A/C) polymorphisms, an elevated risk of cancer in the variant AA genotypes and A allele for the Chinese population was observed. Neither the overall group analyses nor the subgroup analyses stratified by cancer type, including NSCLC, and ethnicity showed a significant association of the rs2280883 and rs3761549 polymorphisms with cancer susceptibility [31]. It appears that the specific genetic variants of FOXP3 and their impact on susceptibility to NSCLC may vary between populations, and ongoing studies may provide more information on this association. Investigating the molecular variants of FOXP3 associated with NSCLC may be helpful in identifying new diagnostic panels.

## 4. FOXP3 Expression in NSCLC

Compared to normal tissues, FOXP3 is highly expressed in various cancers, including gastric cancer, esophageal cancer and breast cancer. The mRNA expression of FOXP3 was significantly increased among NSCLC cell lines (e.g., NCI-H1299, PC9 and NCI-H226) compared to a normal human lung epithelial cell line (BEAS-2B) [27]. In the NSCLC group, FOXP3 is highly expressed in lung squamous cell carcinoma (*p* < 0.001) and lung adenocarcinoma (*p* < 0.001), suggesting that this gene plays a role as an oncogene in lung cancer [8]. The FOXP3 overexpression was confirmed in NSCLC cells and also tumor-infiltrating lymphocytes [32]. In the comprehensive analysis of whole FOXP family mRNA expression, by D. M. Hu et al., the lower *FOXP3* expression was correlated with a better prognosis of lung adenocarcinoma (LUAD) (HR = 1.37 and 95% CI 1.09–1.73) and with a better prognosis of NSCLC (HR = 1.25 and 95% CI 1.1–1.41) [27]. Additionally, the transcriptional level of *FOXP3* was markedly correlated with the six clinicopathological stages in LUAD and lung squamous carcinoma (LUSC) patients. The Kaplan–Meier Plotter showed that the overexpression levels of FOXP2/3 were associated with a poor prognosis of NSCLC [27]. The expression level of FOXP3 was correlated with its promoter methylation level in NSCLC. Methylation involved only one CpGs of the FOXP3 promoter in NSCLC and its level was lower than those in normal samples. A significant prognostic value was demonstrated for FOXP3, as an association was observed between the DNA methylation status of the FOXP3 site and patient survival time [27].

A study by F. Dimitrakopoulos et al. showed that the FOXP3 protein is normally expressed in bronchial epithelial cells but is overexpressed in NSCLC cells and tumor-infiltrating lymphocytes. The lymphocytic FOXP3 expression was an age-related factor, whereas tumor FOXP3 expression was correlated with lymph node metastasis [33]. In another study, the remarkable FOXP3 overexpression in lung adenocarcinoma specimens (N = 40) was confirmed by immunohistochemical (IHC) staining. The increased FOXP3 expression in cancerous tissues was primarily localized in both the nucleus and the cytoplasm of tumor cells [34]. In the study by S. Yang et al., NSCLC patients with the high level of FOXP3 had a significant decrease in overall survival and recurrence-free survival [35]. However, in the study by X. Wang et al., FOXP3 downregulation was found in NSCLC compared with normal tissues, which was a predictor of an unfavorable tumor stage and total and overall survival [36]. In the case of the data extracted from the Human Protein Atlas database, FOXP3 was not expressed in LUSC cancer cells but mainly in immune cells. The result of immunofluorescence (IF) staining verified that FOXP3 colocalized with the T cell marker gene CD3+ in immune cells [8]. A bioinformatics analysis of 1925 patients with NSCLC from the Kaplan–Meier Plotter database indicated 941 patients with high expression of FOXP3 and 984 patients with low expression of this gene. Further, the survival analysis shows that low-expression FOXP3 patients had a median overall survival of 78 months, which was significantly higher than those of high-expression FOXP3 patients (61.21 months). Additionally, a disease-free survival analysis was performed on patients with early stage NSCLC, and the FOXP3 could be used as an indicator of DFS prediction [8].

**Figure 2:**
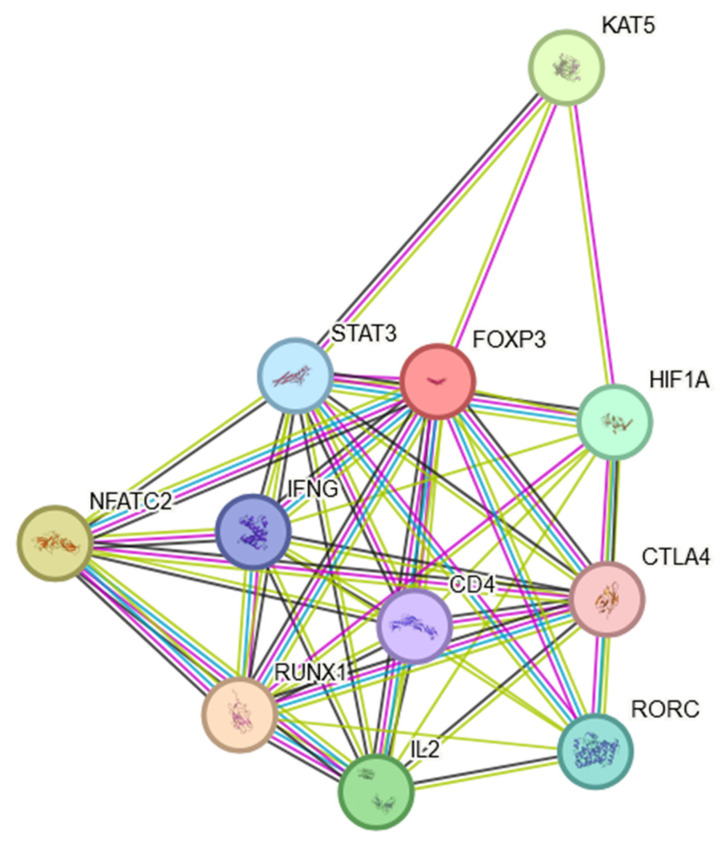
FOXP3 protein STRING interaction network [37].

In conclusion, the expression of FOXP3 in NSCLC is not uniform across all patients or tumor types. Variability in FOXP3 expression may contribute to the diverse outcomes observed in different studies. It was shown that the expression levels of the FOXP3 had significant effects on the clinical parameters, including patient age, smoking habits, histological subtypes, cancer stages, nodal metastasis status and TP53 mutation status. An assessment of FOXP3 mRNA expression levels and an analysis of DNA methylation provides a new approach to the prognosis of NSCLC. It is worth mentioning that the FOXP3 protein is involved in multiple signaling pathways, which may indirectly influence the course of the disease. The FOXP3 protein was found to engage in multiple protein–protein interactions as shown in Figure 2. This STRING interactome [37] indicates that FOXP3 is not only a Treg marker but may also play multiple roles in inflammatory processes present in the TME (Section 5 and Section 6) and NSCLC cancer progression (Section 7 and Section 8), which will be discussed later in this manuscript.

The presented interactome showed that FOXP3 may positively regulate CD4-positive, CD25-positive, alpha-beta regulatory T cell differentiation, T-helper 17 cell differentiation and T cell differentiation. FOXP3 may negatively regulate thymocyte apoptosis.

## 5. Presence of FOXP3 in the TME

**Figure 3:**
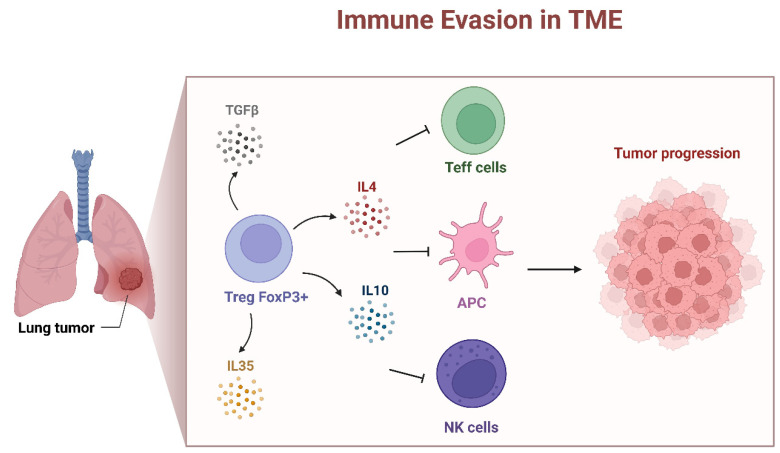
FOXP3+ Tregs mediated immune evasion in tumor environment. FOXP3+ regulatory T cells regulate immune responses by producing inhibitory cytokines, such as TGF-β, IL-4, IL-10 and IL-35, inhibiting T effector cells (Teff), Antigen-Presenting Cells (APCs) and Natural Killer (NK) cells. FOXP3+ Tregs promote an immunosuppressive TME that consequently leads to tumor progression (created with BioRender.com).

FOXP3 is predominantly associated with regulatory T cells [38], and its expression in lung cancer is often linked to the presence of tumor-infiltrating Tregs [32]. It is known that FOXP3 mutations impair Treg cell development and cause IPEX syndrome [25]. The depletion of FOXP3+ CD25+ CD4+ Tregs leads to similar autoimmune diseases in rodents [39]. Lung cancer tumors have been shown to harbor a large number of tumor-infiltrating Treg cells [32]. In patients with NSCLC, it was reported that those with a higher ratio of Tregs to total T cells infiltrating the tumor had an increased risk of recurrence. In addition, increased levels of Tregs in the peripheral blood of NSCLC patients compared to healthy controls was observed [40]. H. Tao et al. indicated that when FOXP3-positive cancer cells were present, the relationship between Treg accumulation and a worse prognosis was attenuated [35]. In the context of NSCLC, the presence of tumor-infiltrating Tregs (FOXP3+ Tregs) can have both positive and negative effects on the immune response [38,41]. The FOXP3+ Tregs are known for their immunosuppressive functions and their presence in the TME may suppress the anti-tumor immune response by the inhibition of the activity of cytotoxic T cells and other effector immune cells, contributing to immune evasion by the cancer cells [38,41] (Figure 3). The occurrence of an immunosuppressive TME promotes tumor growth and progression; for this reason, the FOXP3+ Tregs presence has been associated with a poor prognosis in various solid tumors [42], including NSCLC (HR: 3.91 and *p* < 0.001) [43]. In addition, among patients with node-negative NSCLC (N0), tumor-infiltrating FOXP3+ Tregs were positively correlated with intratumor COX-2 expression and were associated with a worse recurrence-free survival [10].

Tumor-infiltrating Treg cells directly promote tumor immune evasion in multiple mechanisms [42]. The most important is the modulation of expression of checkpoint suppressor molecules, such as CTLA-4 [44], PD-1, TIM-3, LAG-3 and TIGIT [39,41,42] (Figure 2). It was shown that CTLA-4 is constitutively expressed on Treg cells [44]. FOXP3+ Treg cell-mediated immunosuppression is also implemented by the release of a variety immunosuppressive cytokines, e.g., IL-10, IL-35 and TGF-β [42,45]. The mechanisms described above demonstrate that FOXP3+ Treg cells promote immune escape from cancer.

Treg lymphocytes can also strongly suppress cancer-associated inflammation. Generally, Tregs are thought to play a dual role in cancer, as they can suppress anti-tumor immune responses but also inhibit inflammation, which may have both pro- and anti-tumor effects. However, the presence of Tregs with pro-inflammatory features has been reported in NSCLC [46]. Recently, most of the data appreciated that Treg cells present a great phenotypic and functional heterogeneity resulting in distinct Treg cell subsets [47]. It was shown that Treg cells can adopt the transcriptional program and functional characteristics of lineage-specific T effector cells under inflammatory conditions [48]. A number of factors have been described to induce functional instability of Treg cells in the TME, e.g., Treg cell lineage-specific molecules, TCR/CD28 signaling, metabolism and inflammatory cytokines are all factors that have been implicated in the induction of both fragile and former Treg cells in cancer, resulting in the abrogation of the highly immunosuppressive TME and effective control of tumor growth by the immune system [47,48]. The exact mechanisms of this phenomenon have not been fully elucidated.

## 6. Spatial Architecture of Tumor-Infiltrating FOXP3+ T Cells in TME

A growing amount of evidence demonstrates that an intercellular network plays an important role in the regulation of immune cell function in a TME. The development of artificial intelligence resulted in machine learning-based analysis, which is an excellent tool to determine the spatial architecture of a TME.

The machine learning-assisted image analysis was applied to tissue microarrays from 1116 tissue sites of 279 IA-IIIB NSCLS patients, stained by multiplex immunofluorescence for CD8, CD103, PD-1, Tim3, GZMB, CD4, Foxp3, CD31, αSMA, Hif-1α and pan-CK markers [49]. The authors observed a higher density of dysfunctional CD8+ T cells in the invasive margin compared to the tumor center. An evaluation of the mean nearest neighbor distance (mNND) revealed a stronger immunomodulatory network in NSCLC patients with lymph node metastases (LNMs) [49]. The mNND between regulatory CD4+ and intratumoral CD8+ T cells was shorter in patients with LNM compared to those without LNM. The shorter distance between regulatory CD4+ and intratumoral CD8+ T cells was associated with a worse prognosis. In addition, the density of regulatory CD4+ T cells in the tumor center is predominant over those in the invasive margin; however, the difference was not statistically significant [49]. To conclude, the NSCLC patients with lymph node metastases are characterized by the high density of intratumoral-infiltrating CD8+ T cells and more immunosuppressive TME-approaching CD8+ T cells [49].

Multiplex immunofluorescence was also implemented by Peng et al. in 681 NSCLC cases classified from stage IA to IIIB. The analysis indicated close interactions between intrastromal neutrophils and intratumoral Tregs (r^2^ = 0.439 and *p* < 0.001) and intrastromal CD4+ CD38+ T cells and CD20-positive B cells (r^2^ = 0.539 and *p* < 0.001) [50]. In addition, three immune subtypes that were correlated with distinct immune characteristics were identified using the unsupervised consensus clustering approach. The immune-exempted subtype with the highest levels of neutrophils and FOXP+ Tregs was mainly enriched in the advanced stage of NSCLC [50]. Regulatory T cells produce immunosuppressive molecules, such as transforming growth factor (TGF)-β and interleukin (IL)-10 [51,52]. Therefore, the presence of Tregs inhibits the activation and function of CD4+ CD38+ T cells, CD8+ T cells and M1 macrophages and consequently promotes tumor progression. The authors pinpointed intratumoral CD68-positive macrophages, M1 macrophages and intrastromal CD4+ cells, CD4+ FOXP3- cells, CD8+ cells and PD-L1+ cells to be the most robust prognostic biomarkers for DFS in lung cancer [50].

Subsequently, the group of Peng H. et al., utilizing the StarDist depth learning model, determined the spatial location of CD20+ B cells, CD4+ T cells and CD38+ T cells in tissue slices of primary tumors from 553 IA∼IIIB NSCLCs [53]. The distribution of regulatory T cells, marked by FOXP3, was associated with decreased infiltration levels of CD20+ B cells and CD4+ CD38+ T cells (r^2^ = −0.45). In addition, the spatial proximity between the CD66+ neutrophils and FOXP3+ Tregs was associated with decreased infiltrating levels of CD20+ B cells and CD4+ CD38+ T cells (r^2^ = −0.45) [53]. Because CD20+ B cells and CD4+ CD38+ T cells promote cytotoxic T cells for killing tumor cells, the observed distribution pattern indicates that FOXP3+ Tregs and CD66+ neutrophils might synergistically attenuate this process [53]. With an increased number of regulatory T cells and a longer distance between neutrophils and CD20+ B cells, CD4+ T cells were associated with shorter disease-free survival [53].

## 7. FOXP3 Modulates NSCLC Proliferation and Metastatic Potential of NSCLC Cells

FOXP3 overexpression has been shown to facilitate NSCLC invasion and metastasis. However, the underlying mechanism of FOXP3 remains unclear. F. Dimitrakopoulos et al. demonstrated that FOXP3 expression was correlated with lymph node metastasis in patients with NSCLC [33]. In addition, FOXP3 expression was also associated with TNM-stage and lymph node metastasis, which was correlated with the interaction of FOXP3 and TLR4 in tumor cell escape and subsequent tumor progression [54]. It was also demonstrated that FOXP3 promotes tumor growth and metastasis in tumor cells in NSCLC by activating the Wnt/β-catenin signaling pathway [35] and epithelial–mesenchymal transition (EMT) [35,36,55]. The study by S. Yang et al. [35] confirmed that FOXP3 can function as an oncogene in NSCLC. They observed that FOXP3 overexpression significantly induced A549 and NCI-H460 NSCLC cell proliferation, migration and invasion, while its inhibition attenuated its oncogenic function, as confirmed by in vivo studies. FOXP3 increased the luciferase activity of the Topflash reporter and upregulated the Wnt signaling target genes (c-Myc and Cyclin D1) in NSCLC cells. Further co-immunoprecipitation results indicated that FOXP3 interacted with β-catenin/TCF4 to enhance β-catenin and TCF4 function, inducing the transcription of Wnt target genes to promote cell proliferation, invasion and EMT induction [35].

It was also shown that FOXP3 may facilitate the invasion and metastasis of NSCLC cells via regulating VEGF, the EMT and the Notch1/Hes1 pathway [55]. In a study by X. Wang et al., FOXP3 silencing promoted the proliferation, migration and invasion capacity of NSCLC cells and affected the expression level of EMT markers. Furthermore, a marked upregulation of phosphorylated NF-κB was observed in NSCLC cell lines (A549 and H520) after FOXP3 silencing. It was suggested that FOXP3 inhibited NSCLC cell metastasis through NF-κB signaling [36]. Li Cho et al. showed that FOXP3 knockdown significantly inhibited the migratory and invasive capacity of cells and decreased the levels of MMP-2, MMP-9, VEGF, EMT markers (e.g., vimentin and N-cadherin) and Notch1/Hes1. Furthermore, FOXP3 expression was positively associated with CD31+ vascular endothelial cells and negatively correlated with E-cadherin in NSCLC tissues. The study revealed that the pro-metastatic effect of FOXP3 may be linked to the Notch1/Hes1 pathway in NSCLC cells [55].

A novel regulatory axis of MALAT1-FOXP3-GINS1 was unraveled by Li M et al. [56]. The clinicopathological significance of MALAT1 and GINS1 in NSCLC is well known. MALAT1 was shown to mask the protein interaction domain and inhibit FOXP3 ubiquitination by STUB1. The authors showed that MALAT1 plays an important modulatory role in the post-translational modifications of FOXP3, which affects GINS1 transcription and drives the nature of proliferation in NSCLC [56].

## 8. FOXP3 Impact to NSCLC Cancer Stemness

Previously, the expression of FOXP3 has been reported to be associated with the maintenance and survival of stem cells [57]. Q. Haolong et al. found that the smoking carcinogen 4-(N-methyl-N-nitrosamino)-1-(3-pyridyl)-1-butanone (NNK) decreased the expression of EGFR-AS1 in the long term but increased the expression of HIF2A and FOXP3 to stimulate lung cancer cell stemness. In addition, the authors confirmed that the enhancement of lung cancer stemness by FOXP3 was partially stimulated via Notch1 [58]. These results prompted the authors to continue this topic, and in another study, they observed that glioma-associated oncogene homolog 1 (GLI1) promotes the growth of NSCLC by enhancing lung cancer stem cells via stimulating FOXP3 [59]. GLI1 expression was positively correlated with FOXP3. The upregulation of FOXP3 and GLI1 increased the expression lung cancer stem cell markers (e.g., ALDH1A1 and OCT4) and the formation of tumor spheres. In the same study, the involvement of Notch1 activation in the GLI1-mediated FOXP3 pathway was confirmed [59].

## 9. Prognostic Significance of Tumor-Infiltrating FOXP3+ T Lymphocytes

Tumor-infiltrating lymphocytes have crucial prognostic values in solid tumors, considering the ability of the immune response and predicting patients’ response to anticancer therapy. The association between the presence of FOXP3+ CD4+ T cells in TILs has been extensively studied in all subtypes of lung cancer [60,61,62,63].

In patients with stage IA primary LUAD who had undergone surgery, the high levels of FOXP3 TILs were associated with a worse prognosis of overall survival [61]. In addition, CD8-Low/FOXP3-High was significantly associated with a poor prognosis in both overall survival and disease-free survival [61]. The study demonstrated the prognostic value of FOXP3+ and CD8+ for stage IA LUAD. The number of FOXP3+ cells in TILs within the primary lesion was associated with number of extratumoral lymphatic permeation (ly-ext) foci that has been reported as an independent poor prognostic factor for LUAD [62]. The group of Niimi et al. observed a significantly higher number of FOXP+ T cells in LUAD patients with an abundance of ly-ext foci [62]. Hence, an immunosuppressive environment in LUAD might be additionally defined by extratumoral ly-ext foci.

Expression of FOXP3 in tumor-infiltrating lymphocytes in the context of a prognostic marker is extensively studied in NSCLC. The meta-analysis of 45 studies, including 11,448 NSCLC patients, confirmed that FOXP+ TILs in the tumor stroma are poor prognostic markers associated with worse relapse/recurrence-free survival (pooled HR = 1.9) [63]. In contrast, the high densities of CD3+, CD4+, CD8+ and CD20+ in the TILs of the tumor nest are favorable prognostic biomarkers for NSCLC [63].

A strong correlation between the FOXP3 and CD134 (OX40) molecules was observed in I-III NSCLC specimens (rho = 0.691, *p* < 0.0001) [64]. OX40, a member of the TNF receptor family, is involved in T cell co-stimulation and T cell-dependent antibody production [65]. The OX40 receptor suppresses tumor growth by increasing the effector T cell differentiation and proliferation. In addition, CD134 diminished the regulatory T cell activity and as a consequence is a favorable prognostic factor of overall survival in NSCLC [64].

In the stroma of patients with NSCLC, the expression of FOXP3+ tumor-infiltrating lymphocytes was associated with expression of YTHDF1 and YTHDF2 [66]. The human YTH domain family (YTHDF) proteins are RNA-binding proteins that recognize N6-methyladenosine (m6A), facilitating various biological processes via m6A RNA modification [67]. The expressions of both YTHDF1 and YTHDF2 were also independent, favorable prognostic factors for recurrence-free survival (HR, 0.745; 95% CI, 0.562–0.984 for YTHDF1; HR, 0.683; 95% CI, 0.503–0.928 for YTHDF2) [66]; however, the molecular mechanism of acting these molecules in NSCLC is unclear.

The prognostic value and promising clinical application of FOXP3+ was highlighted in SCLC [68]. In TILs, the FOXP3 status was statistically associated with immune markers (CD3, CD4 and CD8) and the immune checkpoint, including PD-1 and PD-L1. This observation, based on an XGBoost machine learning analysis, indicates a strong interaction between FOXP3 and the immune biomarkers in the TME. High FOXP3 expression showed longer relapse-free survival than the low-level group of SCLC (41 months, 95% CI 26.937 to 55.463, vs. 14 months, 95% CI 8.133 to 19.867; *p* = 0.008) [68].

The deep-learning based model was also applied by the group Klein et al. to evaluate different markers of M2 macrophages (CD163 and CD204) together with global immunologic markers (CD4, CD8, CD68, CD38, FOXP3 and CD20) and characterized their intratumoral density in 45 SCLC tumors [69]. The study did not confirm the prognostic relevance of FOXP3. Only the CD163 marker of the M2 macrophages presented significant intratumoral abundance and were associated with an unfavorable outcome in the SCLC cohort. The FOXP3 marker, likewise for CD8, CD4 and CD20, did not reach statistical significance; however, there were increased infiltrates of CD8 and CD20 and a trend for FOXP3-positive cells [69].

## 10. Impact of Cancer Therapy on FOXP3+ TILs

Therapy with immune checkpoint inhibitors, such as nivolumab, pembrolizumab, atezolizumab and durvalumab, has become a standard treatment option for lung cancer, with the TME attracting significant attention [70,71,72,73]. Taking into account the critical role of tumor-infiltrating lymphocytes in the TME, there is an emerging need to identify immune biomarkers of the response to this treatment.

FOXP3 is indicated as one of the promising immune phenotype markers. The immunohistochemistry of CD3+, CD8+, CD68+, CD20+ and FOXP3+ cells from NSCLC treated with ICI revealed that expression of FOXP3+ was associated with shorter progression-free survival (14.8 vs. 1.8 months, *p* = 0.003, HR 8.7 and 95% CI 1.55–48.7) and worse overall survival (22.0 vs. 8.3 months, *p* = 0.035, HR 3.86 and 95% CI 1.01–14.8) [32]. Therefore, the elevated FOXP3+ expression in TILs might be a negative predictive value for (programmed death 1) PD-1 inhibitor therapy in lung cancer [32].

In addition, the TRME2+ macrophages might enhance differentiation toward FOXP3^+^ Tregs, thus facilitating immune evasion of NSCLC [74]. Patients with an elevated number of immunosuppressive TRME2+ macrophages presented a low response rate to anti-PD1 immunotherapy (14.29%) compared to those with low TREM2^+^ macrophages (31.58%) [74].

The limitations of monoclonal antibodies targeting PD-1 and the PD-1 ligand are the cost of producing, impoverished tumor penetration and the risk of autoimmune side effects. A small-molecule antagonist of PD-1/PD-L1 interactions demonstrated potent anti-tumor activity in vitro and in vivo by relieving T cell exhaustion [75]. Incubation of ex vivo activated human T cells with the PDI-1 small molecule enhanced their cytotoxicity toward the human lung by the production of granzyme B, perforin and inflammatory cytokines. PDI-1 decreased the abundance of infiltrating FoxP3+ CD4+ T cells and consequently reduced the growth of the tumor [75]. The small-molecule inhibitors PD-1/PD-L1 hold promise as an alternative or complementary anti-cancer therapeutic agent.

In NSCLC patients carrying an *EGFR* mutation, the EGFR tyrosine kinase inhibitors (EGFR-TKIs) are the front-line treatment. Several phase III studies have demonstrated the superiority of gefitinib, erlotinib (first generation of TKIs) or afatinib (second generation) to chemotherapy in progression-free survival and response rates [76]. It was indicated that EGFR-TKIs impact the tumor immune microenvironment. The density of FOXP3+ TILs was significantly lower after EGFR-TKI therapy than before (249.6→150.4/mm^2^, *p* < 0.0001) [77]. In addition, the changes in the FOXP3+ T cells were related to PD-L1 expression. The FOXP3^+^ TIL density was increased in PD-L1-strong-positive tumor specimens before and after EGFR-TKI treatment compared with the corresponding PD-L1-negative or -low-positive tumor specimens [77]. Therefore, EGFR-TKI therapy complementary with immune checkpoint inhibitors might greatly improve the therapeutic outcome in NSCLC.

Recently published data demonstrated that an intranasal KRAS peptide vaccine is a promising therapeutic approach in *KRAS*-mutated NSCLC [78]. The immunization of a mutant KRAS-mouse lung tumor model resulted in a decrease in CD4^+^FoxP3^+^ T cells in both the lymph nodes and spleen. The cytotoxicity of CD8+ T cells toward KRAS-specific Th1 and Th17 responses was enhanced and persisted 3 months after the last vaccination. The immunized animals had significantly decreased tumor incidence compared to the control animals [78].

## 11. Conclusions

More evidence has recently emerged revealing the carcinogenic effects of FOXP3 in lung cancer. FOXP3 may act as a coactivator to facilitate key signaling pathways, such as WNT, inducing EMT and tumor growth and metastasis in NSCLC. Nevertheless, the prognostic role of FOXP3 needs to be clarified, as it strongly correlates with tumor location, molecular subtype and tumor stage. Overall, understanding the prognostic significance of FOXP3 in NSCLC tumors and its involvement in therapy resistance may help to develop more effective targeted therapeutic strategies to improve clinical outcomes in patients with lung cancer. In addition, it is noteworthy that FOXP3-expressing Tregs play a crucial role in modulating the immune response in lung cancer, promoting tumor immune evasion and contributing to disease progression. Therefore, targeting FOXP3 regulatory T cells may hold promise as a therapeutic approach, and assessing their levels could provide valuable prognostic information for lung cancer patients.

## Data Availability

Not applicable.

## Abbreviations

DFS	disease-free survival
EMT	epithelial–mesenchymal transition
EGFR	epidermal growth factor receptor
EGFR-TKI	EGFR tyrosine kinase inhibitors
FOXP3	forkhead box protein 3
HR	hazard ratio
ICI	immune checkpoint inhibitors
IF	immunofluorescence staining
IHC	immunohistochemical staining
IL	interleukin
IPEX	Immunodysregulation Polyendocrinopathy Enteropathy X-linked
KRAS	Kirsten rat sarcoma virus
LNM	lymph node metastases
LUAD	lung adenocarcinoma
LUSC	lung squamous carcinoma
Ly-ext	extratumoral lymphatic permeation
m6A	N6-methyladenosine
NND	nearest neighbour distance
NSCLC	non-small-cell lung cancer
Nrp-1	neuropilin-1
PD-1	programmed death-1 receptor
PD-L1	programmed death-1 receptor ligand
r2	coefficient of determination
rho	Spearman’s correlation coefficient
SCLC	small-cell lung cancer
SNPs	single-nucleotide polymorphisms
Tregs	regulatory T cells
FoxP3+ Tregs	tumor-infiltrating Tregs
TGF	transforming growth factor
Th1	T helper 1 cell
Th17	T helper 17 cell
TME	tumor microenvironment
TNF	tumor necrosis factor
TRME2	triggering receptor expressed on myeloid cells 2
YTHDF	YTH domain family protein

## References

[1] Sakaguchi S., Sakaguchi N., Asano M., Itoh M., Toda M. (1995). Immunologic self-tolerance maintained by activated T cells expressing IL-2 receptor alpha-chains (CD25). Breakdown of a single mechanism of self-tolerance causes various autoimmune diseases. J. Immunol..

[2] Baecher-Allan C., Brown J.A., Freeman G.J., Hafler D.A. (2001). CD4+CD25high regulatory cells in human peripheral blood. J. Immunol..

[3] Fontenot J.D., Gavin M.A., Rudensky A.Y. (2003). Foxp3 programs the development and function of CD4+CD25+ regulatory T cells. Nat. Immunol..

[4] Brunkow M.E., Jeffery E.W., Hjerrild K.A., Paeper B., Clark L.B., Yasayko S.A., Wilkinson J.E., Galas D., Ziegler S.F., Ramsdell F. (2001). Disruption of a new forkhead/winged-helix protein, scurfin, results in the fatal lymphoproliferative disorder of the scurfy mouse. Nat. Genet..

[5] Bennett C.L., Christie J., Ramsdell F., Brunkow M.E., Ferguson P.J., Whitesell L., Kelly T.E., Saulsbury F.T., Chance P.F., Ochs H.D. (2001). The immune dysregulation, polyendocrinopathy, enteropathy, X-linked syndrome (IPEX) is caused by mutations of FOXP3. Nat. Genet..

[6] Williams L.M., Rudensky A.Y. (2007). Maintenance of the Foxp3-dependent developmental program in mature regulatory T cells requires continued expression of Foxp3. Nat. Immunol..

[7] Honaker Y., Hubbard N., Xiang Y., Fisher L., Hagin D., Sommer K., Song Y., Yang S.J., Lopez C., Tappen T. (2020). Gene editing to induce FOXP3 expression in human CD4(+) T cells leads to a stable regulatory phenotype and function. Sci. Transl. Med..

[8] Zhu J., Li Z., Chen J., Li W., Wang H., Jiang T., Ma Y. (2022). A comprehensive bioinformatics analysis of FOXP3 in nonsmall cell lung cancer. Medicine.

[9] Ohue Y., Nishikawa H. (2019). Regulatory T (Treg) cells in cancer: Can Treg cells be a new therapeutic target?. Cancer Sci..

[10] Shimizu K., Nakata M., Hirami Y., Yukawa T., Maeda A., Tanemoto K. (2010). Tumor-infiltrating Foxp3+ regulatory T cells are correlated with cyclooxygenase-2 expression and are associated with recurrence in resected non-small cell lung cancer. J. Thorac. Oncol..

[11] Tao H., Mimura Y., Aoe K., Kobayashi S., Yamamoto H., Matsuda E., Okabe K., Matsumoto T., Sugi K., Ueoka H. (2012). Prognostic potential of FOXP3 expression in non-small cell lung cancer cells combined with tumor-infiltrating regulatory T cells. Lung Cancer.

[12] Jin Y., Zhang P., Li J., Zhao J., Liu C., Yang F., Yang D., Gao A., Lin W., Ma X. (2015). B7-H3 in combination with regulatory T cell is associated with tumor progression in primary human non-small cell lung cancer. Int. J. Clin. Exp. Pathol..

[13] Suzuki K., Kadota K., Sima C.S., Nitadori J., Rusch V.W., Travis W.D., Sadelain M., Adusumilli P.S. (2013). Clinical impact of immune microenvironment in stage I lung adenocarcinoma: Tumor interleukin-12 receptor beta2 (IL-12Rbeta2), IL-7R, and stromal FoxP3/CD3 ratio are independent predictors of recurrence. J. Clin. Oncol..

[14] Sung H., Ferlay J., Siegel R.L., Laversanne M., Soerjomataram I., Jemal A., Bray F. (2021). Global Cancer Statistics 2020: GLOBOCAN Estimates of Incidence and Mortality Worldwide for 36 Cancers in 185 Countries. CA Cancer J. Clin..

[15] Ferlay J., Colombet M., Soerjomataram I., Parkin D.M., Pineros M., Znaor A., Bray F. (2021). Cancer statistics for the year 2020: An overview. Int. J. Cancer.

[16] Thandra K.C., Barsouk A., Saginala K., Aluru J.S., Barsouk A. (2021). Epidemiology of lung cancer. Contemp. Oncol..

[17] Casal-Mourino A., Ruano-Ravina A., Lorenzo-Gonzalez M., Rodriguez-Martinez A., Giraldo-Osorio A., Varela-Lema L., Pereiro-Brea T., Barros-Dios J.M., Valdes-Cuadrado L., Perez-Rios M. (2021). Epidemiology of stage III lung cancer: Frequency, diagnostic characteristics, and survival. Transl. Lung Cancer Res..

[18] Zhang Y., Vaccarella S., Morgan E., Li M., Etxeberria J., Chokunonga E., Manraj S.S., Kamate B., Omonisi A., Bray F. (2023). Global variations in lung cancer incidence by histological subtype in 2020: A population-based study. Lancet Oncol..

[19] (2023). American Cancer Society: Cancer Facts and Figures 2023. American Cancer Society. https://www.cancer.org/research/cancer-facts-statistics/all-cancer-facts-figures/2023-cancer-facts-figures.html.

[20] Sereno M., Hernandez de Cordoba I., Gutierrez-Gutierrez G., Casado E. (2023). Brain metastases and lung cancer: Molecular biology, natural history, prediction of response and efficacy of immunotherapy. Front. Immunol..

[21] Ferlay J.E.M., Lam F., Laversanne M., Colombet M., Mery L., Piñeros M., Znaor A., Soerjomataram I., Bray F. Global Cancer Observatory: Cancer Today.

[22] Harrison P.T., Vyse S., Huang P.H. (2020). Rare epidermal growth factor receptor (EGFR) mutations in non-small cell lung cancer. Semin. Cancer Biol..

[23] Reita D., Pabst L., Pencreach E., Guerin E., Dano L., Rimelen V., Voegeli A.C., Vallat L., Mascaux C., Beau-Faller M. (2022). Direct Targeting KRAS Mutation in Non-Small Cell Lung Cancer: Focus on Resistance. Cancers.

[24] Chevallier M., Borgeaud M., Addeo A., Friedlaender A. (2021). Oncogenic driver mutations in non-small cell lung cancer: Past, present and future. World J. Clin. Oncol..

[25] Bacchetta R., Barzaghi F., Roncarolo M.G. (2018). From IPEX syndrome to FOXP3 mutation: A lesson on immune dysregulation. Ann. N. Y. Acad. Sci..

[26] Grover P., Goel P.N., Greene M.I. (2021). Regulatory T Cells: Regulation of Identity and Function. Front. Immunol..

[27] Hu D.M., Zhang W.D., Shi Z.E., Zhang M.Y., Li R., Wang Q.X., Ji X.L., Qu Y.Q. (2022). FOXP family DNA methylation correlates with immune infiltration and prognostic value in NSCLC. Front. Genet..

[28] Fazelzadeh Haghighi M., Ali Ghayumi M., Behzadnia F., Erfani N. (2015). Investigation of FOXP3 genetic variations at positions -2383 C/T and IVS9+459 T/C in southern Iranian patients with lung carcinoma. Iran. J. Basic Med. Sci..

[29] He Y.Q., Bo Q., Yong W., Qiu Z.X., Li Y.L., Li W.M. (2013). FoxP3 genetic variants and risk of non-small cell lung cancer in the Chinese Han population. Gene.

[30] Cheng Z., Guo Y., Ming L. (2018). Functional Foxp3 polymorphisms and the susceptibility to cancer: An update meta-analysis. Medicine.

[31] Chen Y., Qi X., Bian C., Ling C., Yi T., Mu X., Zhao X. (2019). The association of FOXP3 gene polymorphisms with cancer susceptibility: A comprehensive systemic review and meta-analysis. Biosci. Rep..

[32] Grell P., Borilova S., Fabian P., Selingerova I., Novak D., Muller P., Kiss I., Vyzula R. (2023). FoxP3 Expression in Tumor-Infiltrating Lymphocytes as Potential Predictor of Response to Immune Checkpoint Inhibitors in Patients with Advanced Melanoma and Non-Small Cell Lung Cancer. Cancers.

[33] Dimitrakopoulos F.I., Papadaki H., Antonacopoulou A.G., Kottorou A., Gotsis A.D., Scopa C., Kalofonos H.P., Mouzaki A. (2011). Association of FOXP3 expression with non-small cell lung cancer. Anticancer Res..

[34] Li Y., Li D., Yang W., Fu H., Liu Y., Li Y. (2016). Overexpression of the transcription factor FOXP3 in lung adenocarcinoma sustains malignant character by promoting G1/S transition gene CCND1. Tumour Biol..

[35] Yang S., Liu Y., Li M.Y., Ng C.S.H., Yang S.L., Wang S., Zou C., Dong Y., Du J., Long X. (2017). FOXP3 promotes tumor growth and metastasis by activating Wnt/beta-catenin signaling pathway and EMT in non-small cell lung cancer. Mol. Cancer.

[36] Wang X., Liu Y., Dai L., Liu Q., Jia L., Wang H., An L., Jing X., Liu M., Li P. (2016). Foxp3 downregulation in NSCLC mediates epithelial-mesenchymal transition via NF-kappaB signaling. Oncol. Rep..

[37] Szklarczyk D., Kirsch R., Koutrouli M., Nastou K., Mehryary F., Hachilif R., Gable A.L., Fang T., Doncheva N.T., Pyysalo S. (2023). The STRING database in 2023: Protein-protein association networks and functional enrichment analyses for any sequenced genome of interest. Nucleic Acids Res..

[38] Dees S., Ganesan R., Singh S., Grewal I.S. (2021). Regulatory T cell targeting in cancer: Emerging strategies in immunotherapy. Eur. J. Immunol..

[39] Tanaka A., Sakaguchi S. (2017). Regulatory T cells in cancer immunotherapy. Cell Res..

[40] Erfani N., Mehrabadi S.M., Ghayumi M.A., Haghshenas M.R., Mojtahedi Z., Ghaderi A., Amani D. (2012). Increase of regulatory T cells in metastatic stage and CTLA-4 over expression in lymphocytes of patients with non-small cell lung cancer (NSCLC). Lung Cancer.

[41] Li C., Jiang P., Wei S., Xu X., Wang J. (2020). Regulatory T cells in tumor microenvironment: New mechanisms, potential therapeutic strategies and future prospects. Mol. Cancer.

[42] Qiu Y., Ke S., Chen J., Qin Z., Zhang W., Yuan Y., Meng D., Zhao G., Wu K., Li B. (2022). FOXP3+ regulatory T cells and the immune escape in solid tumours. Front. Immunol..

[43] O’Callaghan D.S., Rexhepaj E., Gately K., Coate L., Delaney D., O’Donnell D.M., Kay E., O’Connell F., Gallagher W.M., O’Byrne K.J. (2015). Tumour islet Foxp3+ T-cell infiltration predicts poor outcome in nonsmall cell lung cancer. Eur. Respir. J..

[44] Tekguc M., Wing J.B., Osaki M., Long J., Sakaguchi S. (2021). Treg-expressed CTLA-4 depletes CD80/CD86 by trogocytosis, releasing free PD-L1 on antigen-presenting cells. Proc. Natl. Acad. Sci. USA.

[45] Togashi Y., Shitara K., Nishikawa H. (2019). Regulatory T cells in cancer immunosuppression—Implications for anticancer therapy. Nat. Rev. Clin. Oncol..

[46] Phillips J.D., Knab L.M., Blatner N.R., Haghi L., DeCamp M.M., Meyerson S.L., Heiferman M.J., Heiferman J.R., Gounari F., Bentrem D.J. (2015). Preferential expansion of pro-inflammatory Tregs in human non-small cell lung cancer. Cancer Immunol. Immunother..

[47] Hatzioannou A., Boumpas A., Papadopoulou M., Papafragkos I., Varveri A., Alissafi T., Verginis P. (2021). Regulatory T Cells in Autoimmunity and Cancer: A Duplicitous Lifestyle. Front. Immunol..

[48] Koch M.A., Tucker-Heard G., Perdue N.R., Killebrew J.R., Urdahl K.B., Campbell D.J. (2009). The transcription factor T-bet controls regulatory T cell homeostasis and function during type 1 inflammation. Nat. Immunol..

[49] Yang G., Cai S., Hu M., Li C., Yang L., Zhang W., Sun J., Sun F., Xing L., Sun X. (2023). Functional status and spatial architecture of tumor-infiltrating CD8+ T cells are associated with lymph node metastases in non-small cell lung cancer. J. Transl. Med..

[50] Peng H., Wu X., Zhong R., Yu T., Cai X., Liu J., Wen Y., Ao Y., Chen J., Li Y. (2021). Profiling Tumor Immune Microenvironment of Non-Small Cell Lung Cancer Using Multiplex Immunofluorescence. Front. Immunol..

[51] Lambrechts D., Wauters E., Boeckx B., Aibar S., Nittner D., Burton O., Bassez A., Decaluwe H., Pircher A., Van den Eynde K. (2018). Phenotype molding of stromal cells in the lung tumor microenvironment. Nat. Med..

[52] Kim H.R., Park H.J., Son J., Lee J.G., Chung K.Y., Cho N.H., Shim H.S., Park S., Kim G., In Yoon H. (2019). Tumor microenvironment dictates regulatory T cell phenotype: Upregulated immune checkpoints reinforce suppressive function. J. Immunother. Cancer.

[53] Peng H., Wu X., Liu S., He M., Xie C., Zhong R., Liu J., Tang C., Li C., Xiong S. (2023). Multiplex immunofluorescence and single-cell transcriptomic profiling reveal the spatial cell interaction networks in the non-small cell lung cancer microenvironment. Clin. Transl. Med..

[54] Fu H.Y., Li C., Yang W., Gai X.D., Jia T., Lei Y.M., Li Y. (2013). FOXP3 and TLR4 protein expression are correlated in non-small cell lung cancer: Implications for tumor progression and escape. Acta Histochem..

[55] Li C., Wang H., Fang H., He C., Pei Y., Gai X. (2021). FOXP3 facilitates the invasion and metastasis of non-small cell lung cancer cells through regulating VEGF, EMT and the Notch1/Hes1 pathway. Exp. Ther. Med..

[56] Li M., Shi M., Hu C., Chen B., Li S. (2021). MALAT1 modulated FOXP3 ubiquitination then affected GINS1 transcription and drived NSCLC proliferation. Oncogene.

[57] Kasiri S., Shao C., Chen B., Wilson A.N., Yenerall P., Timmons B.C., Girard L., Tian H., Behrens C., Wistuba I.I. (2017). GLI1 Blockade Potentiates the Antitumor Activity of PI3K Antagonists in Lung Squamous Cell Carcinoma. Cancer Res..

[58] Qi H., Wang S., Wu J., Yang S., Gray S., Ng C.S.H., Du J., Underwood M.J., Li M.Y., Chen G.G. (2019). EGFR-AS1/HIF2A regulates the expression of FOXP3 to impact the cancer stemness of smoking-related non-small cell lung cancer. Ther. Adv. Med. Oncol..

[59] Qi H., Li W., Zhang J., Chen J., Peng J., Liu Y., Yang S., Du J., Long X., Ng C.S. (2020). Glioma-associated oncogene homolog 1 stimulates FOXP3 to promote non-small cell lung cancer stemness. Am. J. Transl. Res..

[60] Kotsakis A., Koinis F., Katsarou A., Gioulbasani M., Aggouraki D., Kentepozidis N., Georgoulias V., Vetsika E.K. (2016). Prognostic value of circulating regulatory T cell subsets in untreated non-small cell lung cancer patients. Sci. Rep..

[61] Kinoshita F., Takada K., Yamada Y., Oku Y., Kosai K., Ono Y., Tanaka K., Wakasu S., Oba T., Osoegawa A. (2020). Combined Evaluation of Tumor-Infiltrating CD8+ and FoxP3+ Lymphocytes Provides Accurate Prognosis in Stage IA Lung Adenocarcinoma. Ann. Surg. Oncol..

[62] Niimi T., Nakai T., Aokage K., Tane K., Miyoshi T., Samejima J., Miyazaki S., Taki T., Sakamoto N., Sakashita S. (2022). Prognostic impact of count of extratumoral lymphatic permeation in lung adenocarcinoma and its relation to the immune microenvironment. Cancer Sci..

[63] Chen B., Li H., Liu C., Xiang X., Wang S., Wu A., Shen Y., Li G. (2020). Prognostic value of the common tumour-infiltrating lymphocyte subtypes for patients with non-small cell lung cancer: A meta-analysis. PLoS ONE.

[64] Massarelli E., Lam V.K., Parra E.R., Rodriguez-Canales J., Behrens C., Diao L., Wang J., Blando J., Byers L.A., Yanamandra N. (2019). High OX-40 expression in the tumor immune infiltrate is a favorable prognostic factor of overall survival in non-small cell lung cancer. J. Immunother. Cancer.

[65] Roos A., Schilder-Tol E.J., Weening J.J., Aten J. (1998). Strong expression of CD134 (OX40), a member of the TNF receptor family, in a T helper 2-type cytokine environment. J. Leukoc. Biol..

[66] Tsuchiya K., Yoshimura K., Inoue Y., Iwashita Y., Yamada H., Kawase A., Watanabe T., Tanahashi M., Ogawa H., Funai K. (2021). YTHDF1 and YTHDF2 are associated with better patient survival and an inflamed tumor-immune microenvironment in non-small-cell lung cancer. Oncoimmunology.

[67] Wang X., Zhao B.S., Roundtree I.A., Lu Z., Han D., Ma H., Weng X., Chen K., Shi H., He C. (2015). N(6)-methyladenosine Modulates Messenger RNA Translation Efficiency. Cell.

[68] Jiang M., Wu C., Zhang L., Sun C., Wang H., Xu Y., Sun H., Zhu J., Zhao W., Fang Q. (2021). FOXP3-based immune risk model for recurrence prediction in small-cell lung cancer at stages I-III. J. Immunother. Cancer.

[69] Klein S., Schulte A., Arolt C., Tolkach Y., Reinhardt H.C., Buettner R., Quaas A. (2023). Intratumoral Abundance of M2-Macrophages is Associated With Unfavorable Prognosis and Markers of T-Cell Exhaustion in Small Cell Lung Cancer Patients. Mod. Pathol..

[70] Brahmer J., Reckamp K.L., Baas P., Crino L., Eberhardt W.E., Poddubskaya E., Antonia S., Pluzanski A., Vokes E.E., Holgado E. (2015). Nivolumab versus Docetaxel in Advanced Squamous-Cell Non-Small-Cell Lung Cancer. N. Engl. J. Med..

[71] Garon E.B., Rizvi N.A., Hui R., Leighl N., Balmanoukian A.S., Eder J.P., Patnaik A., Aggarwal C., Gubens M., Horn L. (2015). Pembrolizumab for the treatment of non-small-cell lung cancer. N. Engl. J. Med..

[72] Rittmeyer A., Barlesi F., Waterkamp D., Park K., Ciardiello F., von Pawel J., Gadgeel S.M., Hida T., Kowalski D.M., Dols M.C. (2017). Atezolizumab versus docetaxel in patients with previously treated non-small-cell lung cancer (OAK): A phase 3, open-label, multicentre randomised controlled trial. Lancet.

[73] Antonia S.J., Villegas A., Daniel D., Vicente D., Murakami S., Hui R., Yokoi T., Chiappori A., Lee K.H., de Wit M. (2017). Durvalumab after Chemoradiotherapy in Stage III Non-Small-Cell Lung Cancer. N. Engl. J. Med..

[74] Zhang H., Liu Z., Wen H., Guo Y., Xu F., Zhu Q., Yuan W., Luo R., Lu C., Liu R. (2022). Immunosuppressive TREM2(+) macrophages are associated with undesirable prognosis and responses to anti-PD-1 immunotherapy in non-small cell lung cancer. Cancer Immunol. Immunother..

[75] Wang Y., Gu T., Tian X., Li W., Zhao R., Yang W., Gao Q., Li T., Shim J.H., Zhang C. (2021). A Small Molecule Antagonist of PD-1/PD-L1 Interactions Acts as an Immune Checkpoint Inhibitor for NSCLC and Melanoma Immunotherapy. Front. Immunol..

[76] Hsu W.H., Yang J.C., Mok T.S., Loong H.H. (2018). Overview of current systemic management of EGFR-mutant NSCLC. Ann. Oncol..

[77] Isomoto K., Haratani K., Hayashi H., Shimizu S., Tomida S., Niwa T., Yokoyama T., Fukuda Y., Chiba Y., Kato R. (2020). Impact of EGFR-TKI Treatment on the Tumor Immune Microenvironment in EGFR Mutation-Positive Non-Small Cell Lung Cancer. Clin. Cancer Res..

[78] Wang S.H., Cao Z., Farazuddin M., Chen J., Janczak K.W., Tang S., Cannon J., Baker J.R. (2024). A novel intranasal peptide vaccine inhibits non-small cell lung cancer with KRAS mutation. Cancer Gene Ther..

